# The Synthesis of the Pomegranate-Shaped α-Fe_2_O_3_ Using an In Situ Corrosion Method of Scorodite and Its Gas-Sensitive Property

**DOI:** 10.3390/nano9070977

**Published:** 2019-07-04

**Authors:** Yang Wang, Xincun Tang, Shan Cao, Xi Chen, Zhihao Rong

**Affiliations:** 1 College of Chemistry and Chemical Engineering, Central South University, Changsha 410083, China; 2 School of Light Industry and Engineering, Qilu University of Technology, Shandong 250353, China

**Keywords:** α-Fe_2_O_3_, pomegranate-shaped structure, sensor, scorodite, nanomaterials

## Abstract

The release of hazardous gas increases with the development of industry. The research of gas-sensitive materials has attracted attention. Nanoscale iron oxide (α-Fe_2_O_3_) is one of the research hotspots of gas-sensitive materials because it is a cheap, non-toxic semiconductor material. In this study, pomegranate-shaped α-Fe_2_O_3_ was synthesized using an in situ corrosion method of scorodite. Spherical-shaped α-Fe_2_O_3_ nanoparticles were included in the octahedral shells. The forming process of the structure was analyzed by a variety of measurements. The shell was formed first through the deposition of Fe(OH)_3_, which was produced by hydrolyzing scorodite. Then, the corrosion was continued and Fe(OH)_3_ precipitation was produced below the shell. The particles aggregated and formed spheres. The pomegranate-shaped α-Fe_2_O_3_ was formed when the scorodite was hydrolyzed completely. The gas-sensing properties of α-Fe_2_O_3_ were investigated. The results showed that pomegranate-shaped α-Fe_2_O_3_ was responsive to a variety of gases, especially xylene. The value of R_a_/R_g_ was 67.29 at 340 °C when the concentration of xylene was 1000 ppm. This indicated the pomegranate-shaped α-Fe_2_O_3_ has potential application as a xylene gas sensor.

## 1. Introduction

With the continuous advancement of science and technology, the emissions of toxic gas have become increasingly larger, and the number of industrial pollution and gas poisoning accidents has gradually increased [1,2]. Therefore, many researchers are committed to the preparation of highly dispersible, porous, large surface area inorganic metal oxide materials which are applied in gas sensors [3,4]. This has become a recent research focus of researchers. The most widely used gas sensor is a semiconductor gas sensor, which converts gas species and their concentration-related information into electrical signals [5,6]. These sensors can be used to detect flammable, explosive, and toxic gas, for example, carbon monoxide, hydrogen sulfide, and sulfur dioxide, and ethanol [7,8,9]. Xylene mainly comes from chemical engineering materials, such as painting and adhesive in indoor environments. Xylene can cause tremendous damage to human beings even in microscale amounts [10]. In addition, careless oral ingestion of xylene can cause acute pneumonia and cancer. Thus, detecting the presence of xylene is very significant [11]. The selective detection of ppm-level xylene using oxide semiconductor remains a challenging issue [10]. Semiconductor gas sensors have a wide measurement range, high sensitivity, fast response recovery, long life, and especially low cost and easy fabrication [12]. They have been widely used and have great application prospects in the field of monitoring organic volatile gas [13].

Nanoscale iron oxide (α-Fe_2_O_3_) is one of the research hotspots of gas-sensitive materials because it is a cheap, non-toxic, and wide band gap semiconductor material [14,15]. α-Fe_2_O_3_ is a N-type semiconductor, and the electrical conductivity of α-Fe_2_O_3_ is very sensitive such that it can be used as a highly efficient gas-sensing material [16,17]. In addition, α-Fe_2_O_3_ has certain chemical stability and thermal stability, which ensures its reproducibility as a gas-sensitive material [18]. Nowadays, the methods for synthesizing α-Fe_2_O_3_ nanomaterials mainly include hydrothermal method, solvothermal method, template method, and common liquid phase method [19,20,21,22]. Generally, the synthesized nano α-Fe_2_O_3_ are nanospheres, nanorods, and nanosheets [23,24,25]. Their structures are usually loose and have a small specific surface area [26]. These factors limit the application of iron oxide gas-sensitive materials.

In this study, the pomegranate-shaped α-Fe_2_O_3_ was synthesized using an in situ corrosion method of scorodite. As shown in Figure S1 (Supplementary Materials), the pomegranate shape meant a large number of spheres were encased in a shell which consisted of nanoparticles. Spherical-shaped α-Fe_2_O_3_ nanoparticles were included in the octahedral shells. This paper was the first to report the pomegranate structure in the nano α-Fe_2_O_3_ research area. The forming process was simple and easy to operate. The synthesized pomegranate-shaped α-Fe_2_O_3_ was detected by X-ray powder diffraction (XRD), X-ray photoelectron spectroscopy (XPS), energy dispersive spectrometry (EDS), scanning electron microscopy (SEM), transmission electron microscopy (TEM), and Brunauer–Emmett–Teller (BET) method. The gas-sensing property of the α-Fe_2_O_3_ was investigated. The results showed the pomegranate-shaped α-Fe_2_O_3_ was responsive to a variety of gases, especially xylene. This indicates the pomegranate-shaped α-Fe_2_O_3_ is a new xylene-sensing material with potential application prospects. 

## 2. Materials and Methods

### 2.1. Reagents

Sodium arsenate (Na_3_AsO_4_·12H_2_O, Sinopharm Chemical Reagent Co., Ltd, Shanghai, China) was used as the As(V) source. Ferrous sulfate heptahydrate (FeSO_4_·7H_2_O, Sinopharm Chemical Reagent Co., Ltd, Shanghai, China) was used as the iron source. Sulfuric acid (H_2_SO_4_, 98%, Sinopharm Chemical Reagent Co., Ltd, Shanghai, China) was used only for pH adjustment. The alkaline soaking solution was sodium hydroxide (NaOH, Sinopharm Chemical Reagent Co., Ltd, Shanghai, China). All of these chemicals were analytical grade. Compressed oxygen gas (O_2_, purity 99.9%, Gaoke special gas Co., Ltd, Changsha, China) was employed as an oxidizing agent. 

### 2.2. Synthesis 

The scorodite precursor was prepared by atmospheric method [27,28]. The specific process is described in the Supporting Information. Aqueous alkaline solution was prepared by dissolving NaOH (pH = 14). The synthesized scorodite was added into the NaOH solution. After reacting for 30 min, the precipitation was filtered and washed with distilled water. The precipitation was calcined at different temperatures with a heating rate of 10 °C/min for 1 h in air. The products were pomegranate-shaped α-Fe_2_O_3_. 

The sample calcined at 500 °C was named S1. The sample without thermal treatment was named S2. The sample which was reacted for 5 min in the NaOH solution without thermal treatment was named S3. 

### 2.3. Characterization

The morphology of synthesized products was observed by field emission scanning electron microscopy (SEM, S-4800, Hitachi, Tokyo, Japan) and high-resolution transmission electron microscopy transmission electron microscope (HRTEM) with an accelerating voltage of 200 kV (JEM-2100, JEOL, Tokyo, Japan). The component element and content of the products were measured by energy disperse X-ray spectroscopy (EDS) on EDAXTLS attachment with an operating voltage of 30 kV (S-4800, Hitachi, Tokyo, Japan). X-ray photoelectron spectroscopy (XPS) spectra were recorded (ESCALAB 250 Xi, Thermo Fisher Scientific, Waltham, MA, USA). The XRD patterns were collected using a Bruker D8 diffraction instrument with Cu Kα radiation (40 kV, 40 mA) (D8, Bruker, Bremen Germany). Surface area quantification was achieved using the Brunauer–Emmett–Teller (BET) method on a Micromeritics ASAP 2020 instrument using nitrogen gas, and pore size distribution was obtained according to the Barrett–Joyner–Halenda (BJH) algorithm (ASAP 2020, Micromeritics, Norcross, GA, USA). 

### 2.4. Gas-Sensing Test 

The gas sensor was made in a conventional manner. Briefly, the prepared product was dispersed in terpineol, which was used as a binder. An alumina ceramic tube assembled with a platinum wire electrode for electrical contacts is immersed in the slurry multiple times to form the sensing film. The Ni–Cr alloy wire, as a resistance heater, was then passed through the ceramic tube. To improve stability, the sensor was aged in air at 200 °C for 3 days before the test. The test was conducted in the commercial gas-sensing measurement system WS-30A (Wensen Electronic Technology Co., Ltd. Zhengzhou, China). The relative humidity can affect the sensing property of the materials. The water molecules can change the resistance of the materials. In this study, the relative humidity was set at 10% ± 1% as controlled by the gas-sensing measurement system.

Response of a sensor was defined as follows:

(1) $$\mathrm{Response}=\frac{R_{a}}{R_{g}}$$

R_a_ is the resistance of the sensor in the air, and R_g_ is the mixture of the test gas and air. Response time is defined as the time required for the conductance to reach 90% of the equilibrium value after injection of the test gas, and the recovery time is the time required for the sensor to reach 10% of the conductance compared to its original value in air. The calculation values of conductance used were the conductance of samples in the air. The gas-sensitive test was a dynamic responses process. The calculation of time was started from the filling of xylene into the test chamber. The purification process was that the mixture gas was exhausted by a powerful fan. The xylene was refilled after the purification step. The whole process was dynamically continuous and repeated several times. The value of response was the average value of the whole test process.

## 3. Results and Discussion

### 3.1. Characterization of Scorodite Precursor 

**Figure 1:**
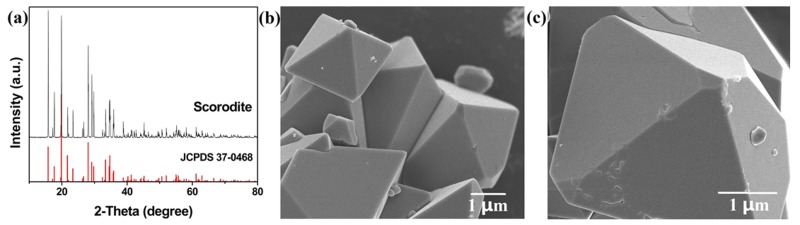
(**a**) The XRD pattern and (**b**,**c**) SEM images of as-synthesized scorodite.

The scorodite precursor was prepared by atmospheric method. As shown in Figure 1a, the pure scorodite was successfully synthesized. The main peaks of scorodite were located at approximately 19.8°, 28.0°, and 15.8°, which were indexed as the (200), (212), and (111) lattice planes of scorodite (JCPDS No. 37-0468) [29]. The diffraction peak intensity was high, which indicated the scorodite had excellent crystallinity. SEM images show the morphology of scorodite crystals (Figure 1b,c). The scorodite crystals were regular octahedrons, with a size of about 5 μm. The surface of scorodite particles was smooth.

### 3.2. Characterization of Samples 

**Figure 2:**
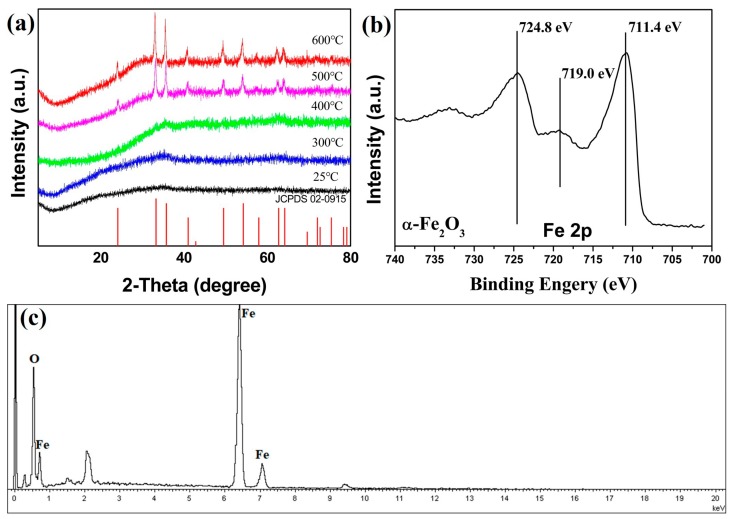
(**a**) The XRD pattern of products by calcined at different temperature, (**b**) XPS spectrum, and (**c**) EDS spectrum of α-Fe_2_O_3_ which was prepared by calcination at 500 °C.

The synthesized scorodite precursor was added in the pH = 14 NaOH solution. After reacting for 30 min, the precipitation was filtered and washed with distilled water. Then, the precipitation was calcined at different temperatures with a heating rate of 10 °C/min for 1 h in air. After cooling to room temperature, the samples were detected by XRD. Figure 2a shows the XRD patterns of products by calcined at different temperatures of 300, 400, 500, and 600 °C. Scorodite can hydrolyze in the high pH alkaline solution. Compared with Figure 1a, the characteristic peaks of scorodite disappeared completely. The results indicated that all the scorodite was transformed into amorphously formed compounds by the corrosion of OH^−^, and that the product before calcination was Fe(OH)_3_, according to the following reaction:

FeAsO_4_·2H_2_O + OH^−^ → Fe(OH)_3_ + AsO_4_^3−^ + 2H^+^ (2)

When the precipitation was calcined at 300 and 400 °C, the XRD patterns had little change at ~33°. After being calcined at 500 and 600 °C, fully hematite phase α-Fe_2_O_3_ was obtained (JCPDS No.02-0915) [30]. There were no other characteristic peaks. It indicated the products were pure α-Fe_2_O_3_. The reaction can be represented as follows:

2Fe(OH)_3_ → α-Fe_2_O_3_ + 3H_2_O (3)

In order to further determine the product which was calcined at 500 °C, XPS and EDS measurements were made. As shown in Figure 2b, the binding energy values of the Fe_2p_ signal in the scorodite samples were 711.4 and 724.8 eV, which correspond to Fe_2p3/2_ and Fe_2p1/2_, respectively. The energy difference between Fe_2p3/2_ and Fe_2p1/2_ peaks was 13.4 eV. Furthermore, Fe^3+^ satellite peak was observable in the spectrum at 719.0 eV, above the Fe_2p3/2_ peak. The results of XPS were consistent with the previously reported for α-Fe_2_O_3_ [31]. The EDS test indicated that arsenic element was non-existent, and that there were only iron and oxygen elements. The results demonstrated the conversion of scorodite to Fe(OH)_3_ after soaking in a pH = 14 NaOH solution. The decomposition reaction was occurred by calcined, and the Fe(OH)_3_ converted to α-Fe_2_O_3_. The sample calcined at 500 °C was named S1. The sample without thermal treatment was named S2. 

### 3.3. Morphology of S1 and S2

**Figure 3:**
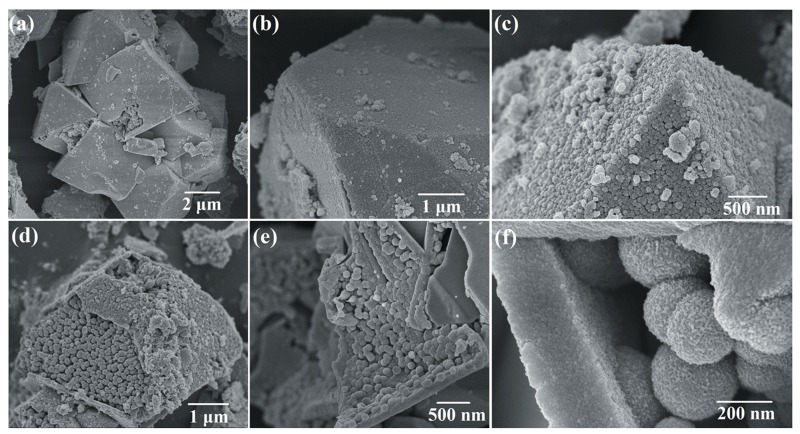
The SEM images of (**a**,**b**) S1 and (**c**) S2, the SEM images of (**d**,**e**) S1, (**f**) S2 treated by ultrasonic.

Figure 3a–c shows the morphology of S1 (calcined at 500 °C) and S2 (without thermal treatment). The octahedral structure remained the same as the scorodite. However, the smooth surface became a shell composed of small particles. The high-magnification SEM image of the surface for S1 can be found in Supplementary Materials. The small particles could obviously be observed (Figure S3). Some spheres with diameter of ~200 nm particles were observed through the breakage of octahedral shell. The particles of S1 was smaller than S2 because the amorphous Fe(OH)_3_ lost H_2_O molecular, and converted to α-Fe_2_O_3_. The size of particles reduced. The compactness of S1 was increased compared with S2. The reason was that the particles aggregated together through the thermal treatment. In order to examine the morphology of particles inside, S1 and S2 were treated by ultrasonic to break the shell. As shown in Figure 3d–f, the shell was destroyed, and the structure inside of S1 and S2 could be clearly observed. The structures of S1 and S2 were both pomegranate-shaped nano–microstructure. The structure of S1 was that of α-Fe_2_O_3_ spheres (~200 nm) were coated by an octahedral shell which made up of nanoparticles. The structure of S2 was spheres (~250 nm) coated by an octahedral shell. The thickness of shell for S1 is about 200 nm. The results of SEM images indicated the scorodite was completely hydrolyzed in the pH = 14 NaOH solution after 30 min. However, the octahedral structure remained because the corrosion reaction by OH^−^ was mild and slow, and the structure of scorodite was not damaged. The pomegranate-shaped structure also remained after the thermal treatment because of the maintenance of structure.

**Figure 4:**
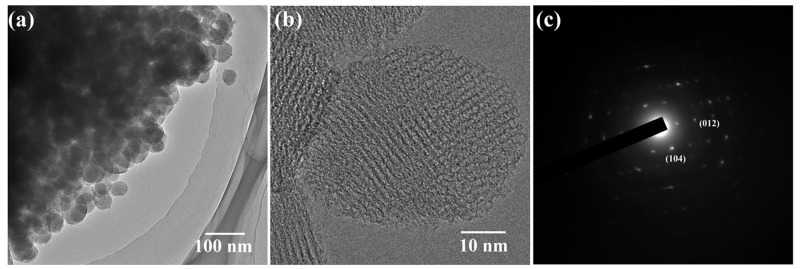
(**a**,**b**) The TEM images of S1 and (**c**) the SAED pattern of S1.

By TEM analysis, the morphology of S1 (calcined at 500 °C) is of nanospheres. The results of Figure 4a were consistent with the results of SEM test. As shown in Figure 4b, the lattice fringes were clear and the lattice spacings were 0.27 and 0.37 nm, corresponding to the (104) and (012) lattice planes, respectively (Figure 4c) [32]. This closely matched the results obtained from the XRD diagram in Figure 2a and verified that S1 was crystalline α-Fe_2_O_3_.

### 3.4. Analysis of the Forming Process of the Pomegranate Structure

To analyze the forming process of the pomegranate shape structure, the reaction in NaOH solution was stopped at 5 min by filtering and washing with deionized water. The product was named S3.

**Figure 5:**
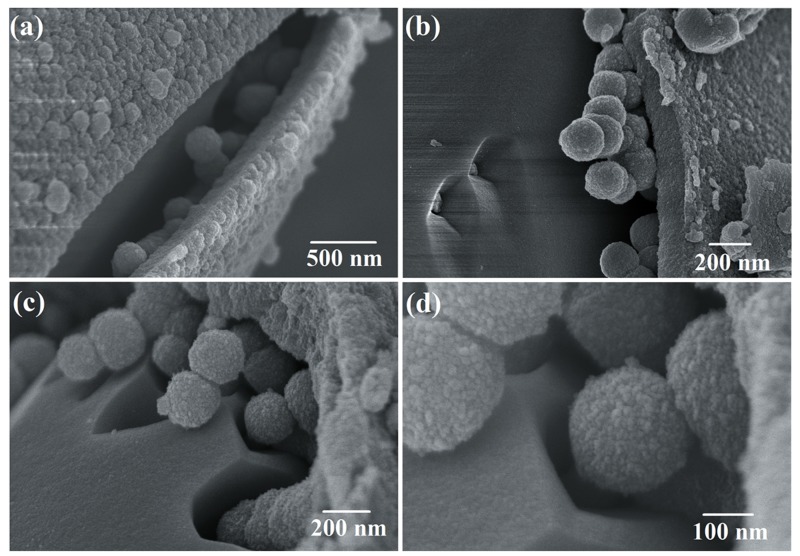
The SEM images of (**a**,**b**) S3 and (**c**,**d**) the detail of S3 treated by ultrasonic.

Figure 5 shows the morphology of S3. Through the results of SEM images, the scorodite crystals observed, which indicated the scorodite was not completely hydrolyzed when the reaction time was 5 min. As shown in Figure 5a,b, a shell consisting of small particles was formed first. The surface of scorodite (FeAsO_4_·2H_2_O) was corroded by OH^−^ ions in the alkaline solution, and hydrolysis reaction of FeAsO_4_·2H_2_O occurred. Fe(OH)_3_ precipitation was produced and deposited on the surface of the scorodite crystals. With the deposition and growth, the Fe(OH)_3_ shell was formed. Figure 5c,d show the forming process of spheres inside. The external alkaline liquid can penetrate internally through the interstices of the shell. The internal scorodite crystal was continuously corroded, and Fe(OH)_3_ particles were produced. However, the existence of the shell limited the diffusion of Fe(OH)_3_. Due to the principle of minimum surface energy, these particles aggregated and formed spheres below the shell [33]. The spherical structure could decrease the surface energy, making the overall material more stable. Finally, scorodite (FeAsO_4_·2H_2_O) was completely corroded by OH^−^ and formed spheres with a diameter of ~200 nm.

### 3.5. BET Analysis of S1

**Figure 6:**
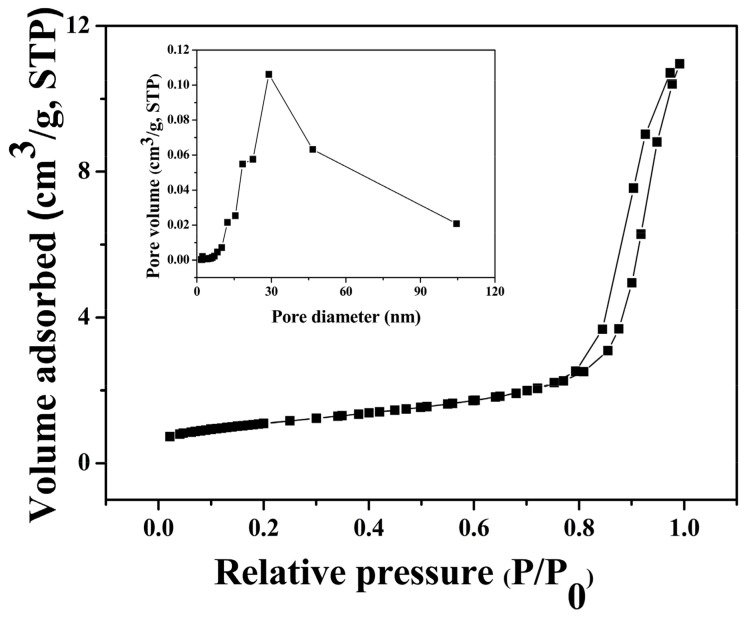
Nitrogen adsorption–desorption isotherms and the corresponding BJH pore size distributions (insets) of S1.

For the metal oxide-based sensors, the gas-sensing mechanism involves an adsorption–oxidation–desorption process. The behavior of the sensor is based on changes in resistance during the process. Due to the important effect of specific surface area on the gas sensitivity, nitrogen adsorption–desorption measurement of S1 was conducted. Figure 6 shows nitrogen adsorption–desorption isotherms and the corresponding pore size distribution curves (inset of Figure 6) of S1. It exhibited type IV adsorption–desorption isotherms curves, as previously reported [34]. The pore size distribution from Barrett–Joyner–Hallenda (BJH) method (inset of Figure 6) demonstrates that S1 was made up of mesoporous structures and the specific surface area of S1 was determined to be 88.45 m^2^/g. This is larger than the previous reports of 16.02, 33, and 45 m^2^/g [35,36].

### 3.6. Gas-Sensitive Properties of Samples

**Figure 7:**
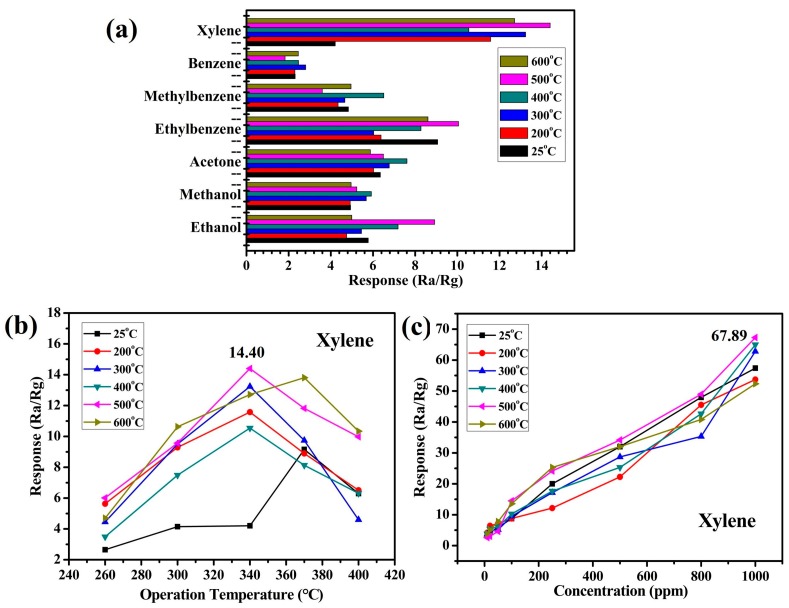
(**a**) Responses of the sensors to 100 ppm various vapors at 340 °C, (**b**) relationship between working temperature and response to 100 ppm xylene, and (**c**) relationship of the concentration of xylene versus response of the sensors at 340 °C. The lines of different color in Figure 7 represent the samples which were calcined by different temperatures. The 340 °C in Figure 7a–c and the abscissa of Figure 7b represent the operation temperature in gas-sensitive tests.

It is common knowledge that the selectivity is an important indicator of gas-sensitive sensors in actual applications. Figure 7a shows the responses of the synthesized samples which were calcined at different temperature for various organic vapors, such as xylene, benzene, methylbenzene, ethylbenzene, acetone, methanol, and ethanol, with a concentration of 100 ppm at 340 °C. This showed that the synthesized pomegranate-shaped α-Fe_2_O_3_ had certain response values to these gases, and that the response value to xylene was higher than with the other gases. The sample which was calcined at 500 °C was found to be the best (S1). Xylene is toxic and dangerous to the environment and humans due to its flammability and carcinogenicity [11]. The results of Figure 7a indicate the pomegranate-shaped α-Fe_2_O_3_ has potential as a xylene gas-sensitive material. Figure 7b,c show the gas-sensitive properties of pomegranate-shaped α-Fe_2_O_3_ to xylene at different working temperatures or in different concentration environments. The largest value of R_a_/R_g_ was 14.40 when the working temperature was 340 °C. The samples which were calcined over 200 °C would be stable during the aging and gas-sensitive tests. However, S2 (25 °C, without thermal treatment) should be convert to another form during the aging process. Hence, it showed an irregular and large fluctuation when the operation temperature of the gas-sensitive test was changed. Sample S1 was the best sample when the concentration of xylene was 100 ppm at 340 °C. As shown in Figure 7c, the responses of S1 were more sensitive than the other samples under different concentrations of xylene. The largest value of R_a_/R_g_ was 67.89 when the concentration of xylene was 1000 ppm. This result is better than the value of 17.54 in a previous study [37]. In the case of metal oxide-based sensors, the gas-sensing mechanism involves an adsorption–oxidation–desorption process. The adsorbed O_2_ on the surface of the sensor, which trap electrons from the bulk, will transform to the O_2_^−^, O^−^, and O^2−^ ions. When a reducing gas such as xylene is introduced, the interaction of oxygen ions with xylene will happen. The electrons produced in the oxidation reaction then go back to the bulk. The behavior of sensor is based on changes of electric resistance in the whole process [38,39]. Here, xylene may undergo the following reaction on the surface of the α-Fe_2_O_3_ sensors:

C_6_H_4_(CH_3_)_2_ + 21O^−^ → 8CO_2_ + 5H_2_O(g) + 21e^−^ (4)

The results indicate the pomegranate-shaped α-Fe_2_O_3_ had an excellent gas-sensitive property for detecting xylene. The outer shell constrained the internal sphere particles, resulting in the material having a relatively large density. The structure could provide larger specific surface area and more active sites for contact with gas molecules. The pomegranate-shaped α-Fe_2_O_3_ has potential for application as a xylene gas sensor.

## 4. Conclusions

The pomegranate-shaped α-Fe_2_O_3_ was synthesized using an in situ corrosion method of scorodite. Spherical-shaped α-Fe_2_O_3_ nanoparticles were included in the octahedral shells. The structure in this study was the first report in the nano α-Fe_2_O_3_ research area. The forming process of the structure was analyzed by a variety of measurements. The shell was formed first through the deposition of Fe(OH)_3_, which was produced by hydrolyzing scorodite. Then, the corrosion was continued and Fe(OH)_3_ precipitation was produced below the shell. The particles aggregated and formed spheres because of the principle of minimum surface energy. Pomegranate-shaped α-Fe_2_O_3_ was formed when scorodite was completely hydrolyzed. The gas-sensing properties of α-Fe_2_O_3_ were investigated. The results showed pomegranate-shaped α-Fe_2_O_3_ was responsive to a variety of gases, especially xylene. The value of R_a_/R_g_ was 67.29 at 340 °C when the concentration of xylene was 1000 ppm. This indicated the pomegranate-shaped α-Fe_2_O_3_ has potential application as a xylene gas sensor.

## Supplementary Materials

The following are available online at https://www.mdpi.com/2079-4991/9/7/977/s1, Figure S1: the schematic diagram of pomegranate shape, Figure S2:.the XRD pattern of as-synthesized scorodite, Figure S3: the high magnification SEM image of the surface for S1.
