# Supplementary material for: The Synthesis of the Pomegranate-Shaped α-Fe2O3 Using an In Situ Corrosion Method of Scorodite and Its Gas-Sensitive Property
Source: Nanomaterials (Basel). 2019 Jul 4;9(7):977. doi: 10.3390/nano9070977 (PMC6669738; doi:10.3390/nano9070977)
Supplement: Supplementary file 1 [file nanomaterials-09-00977-s001.pdf]

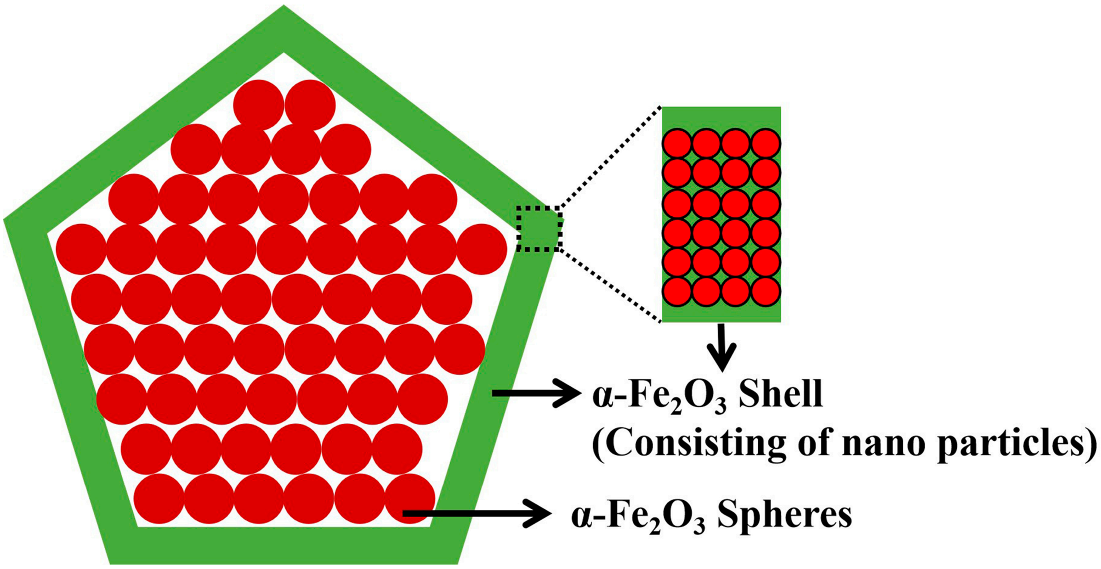

Figure S1. the schematic diagram of pomegranate shape.

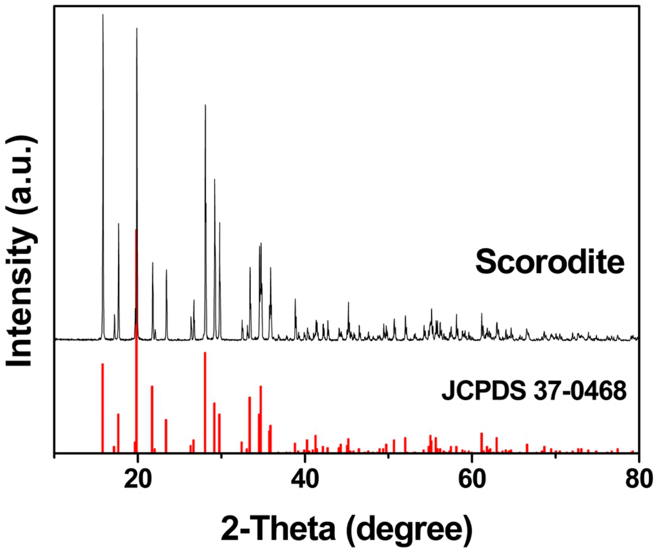

Figure S2. the XRD pattern of as-synthesized scorodite

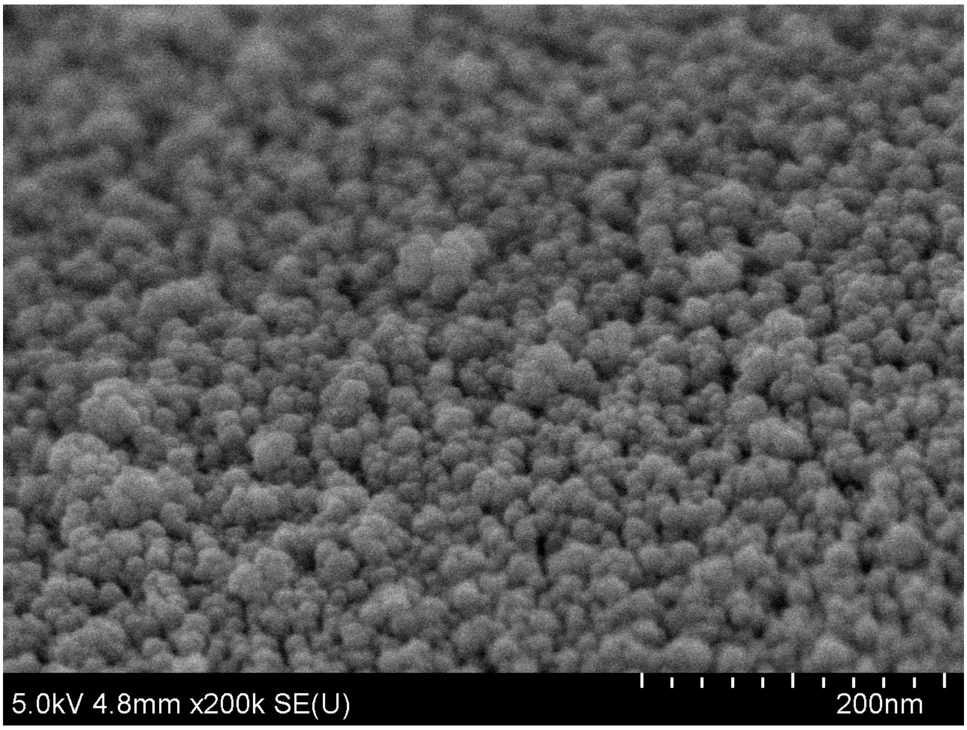

Figure S3. the high magnification SEM image of the surface for S1

1. Synthesis of scorodite

The scorodite was prepared by atmospheric method. Highly purity O_2 was injected into the aqueous solution which included As(V) and Fe(II) ions at 90 °C. 20 g/L aqueous As(V) solution was prepared from the dissolution of $Na_3AsO_4\cdot 12H_2O$, and pH of the solution was adjusted 1.0 by 98 % H_2SO_4. The As(V) solution with 20 g/L As(V) concentration was reacted with $FeSO_4\cdot 7H_2O$ in a three-necked flask, and the molar ratio of Fe(II) and As(V) ions was 3:2. The solution was heated by oil bath and stirred at 1000 rpm until the system achieved the reaction temperature (90 °C). Afterwards, highly purity O_2 was injected into the solution, reacting for 7 hours at 90 °C. Then the suspension was filtered and washed with deionized water, and dried in an oven at 70 °C to obtain the scorodite.
